# Management of Diabetic Ketoacidosis in a Patient With Chronic Kidney Disease Under Maintenance Hemodialysis: A Case Report

**DOI:** 10.7759/cureus.42700

**Published:** 2023-07-30

**Authors:** Milan Regmi, Anurag Karki, Sanjeev Bhandari, Moon Shrestha, Pooja Kafle

**Affiliations:** 1 Internal Medicine, Tribhuvan University Institute of Medicine, Kathmandu, NPL; 2 Critical Care Medicine, Tribhuvan University Institute of Medicine, Kathmandu, NPL

**Keywords:** electrolyte in dka, fliud management in dka, insulin therapy, maintenance hemodialysis, chronic kidney disease, diabetic ketoacidosis

## Abstract

Diabetic ketoacidosis (DKA) is a potentially fatal metabolic complication seen in individuals with type 1 diabetes mellitus (DM) or type 2 DM under stress, such as infections and non-compliance with treatment. DKA in chronic kidney disease (CKD) patients undergoing maintenance hemodialysis (HD) presents challenges due to the unique pathophysiology and the absence of specific management guidelines. This case report highlights the importance of tailoring the treatment of DKA based on the specific requirements of CKD patients on HD. The presented case involves a 47-year-old female with type 2 DM and CKD who developed DKA in the context of a urinary tract infection (UTI). Management included insulin infusion, cautious fluid replacement therapy, electrolyte monitoring, and identifying precipitating factors, such as an infection. The case highlights the complexity of DKA management in CKD patients and the necessity of individualized approaches. More studies and guidelines are needed to optimize the proper management of DKA in CKD patients.

## Introduction

Diabetic ketoacidosis (DKA) is a common and potentially fatal metabolic abnormality in insulin-dependent diabetes mellitus (DM) [1,2]. DKA occurs commonly in type 1 DM but can occur in type 2 DM under stressful conditions, such as infections, noncompliance to treatment, and insulin omission, of which infections are the most common precipitating factor [2-4]. Among all cases of DKA, about one-third are due to type 2 DM [5]. The management of DKA in the general population is established, but in a patient with renal failure under maintenance hemodialysis (MHD), no specific guidelines are available [6-8]. There are marked differences in the pathophysiology of DKA in a patient with chronic kidney disease (CKD) from DKA in the general population with normal renal function. Therefore, the management of DKA in CKD should differ and be tailored accordingly from established guidelines for appropriate management [9]. Moreover, a patient with DKA and CKD under MHD has a higher incidence of volume overload, hypoglycemia, longer stay, and need for ventilation than a patient with normal renal function [10]. 

Very few articles are related to managing DKA in a patient with CKD. Even in tertiary centers, we get to see very few cases. Our case report highlights the importance of calibrating treatments according to the need of patients with CKD under MHD.

## Case presentation

A 47-year-old female diagnosed with type 2 DM, hypertension, and CKD secondary to chronic glomerulonephritis presented to the emergency department with bilateral swelling of lower limbs for 15 days, nausea for five days, and altered sensorium for one day. She was on MHD twice weekly for the last four years. She was diagnosed with type 2 DM 20 years back and was under metformin for the first 11 years, followed by insulin therapy for the past nine years. Her fasting blood sugar from three months prior was 9.3 mMol/L, indicating that her diabetes was not under control. She is hypertensive, has been under medication for 12 years, and has had a history of hypothyroidism for the past two years, for which she has been taking levothyroxine. She had her last hemodialysis (HD) session three days before the presentation. She had no history of missed insulin doses.

Her Glasgow Coma Scale (GCS) was 12/15, E4, V4, and M4 on examination. Vitals showed a pulse of 84 beats/min, a respiratory rate of 22 breaths/min, oxygen saturation maintained at room air, and a blood pressure of 160/90 mmHg. Bilateral pitting pedal edema was present on general examination, whereas systemic examination showed no significant findings. In addition, she had no flapping tremors. 

Her investigations on the day of admission and two days after are depicted in Table 1. On the day of admission, her blood glucose was 27.1 mmol/L, creatinine was 718 μmol/L, and urea was 35 mmol/L. Hematological investigation shows hemoglobin of 9.7 g/dL with normal total leukocyte and platelet counts. Arterial blood gas (ABG) showed pH of 7.2 with HCO_3_ 6 mmol/L, PCO_2_ 15.2 mmHg, K+ 3.5 mEq/L, Na 128 mEq/L, and lactate 9.2 mmol/L (normal 0.5-2.2 mmol/L). Her urine analysis was positive for acetone and albumin 3+, and plenty of red blood cells (RBCs) and pus cells were seen. Reverse transcription polymerase chain reaction (RT-PCR) for severe acute respiratory syndrome coronavirus 2 (SARS‑CoV2) was negative. Electrocardiogram (ECG) was normal, and chest X-ray showed no signs of pulmonary edema or pneumonia. Urine culture of clean catch midstream urine was positive for *Escherichia coli*, with >10^5^ colony forming units (CFUs), indicating a urinary tract infection (UTI), whereas blood culture was negative for *E. coli*. 

**Table 1:** Laboratory parameters on the day of admission and two days after admission (with normal reference range)

Parameters	On the day of admission	Two days after admission	Normal range (units)
Urea	35 mmol/L	16 mmol/L	2.8-7.2 mmol/L
Creatinine	718 μmol/L	482 μmol/L	45-84 μmol/L
Sodium	128 mEq/L	135 mEq/L	135-146 mEq/L
Potassium	3.5 mEq/L	3.5 mEq/L	3.5-5.2 mEq/L
pH	7.2	7.46	7.38-7.44
Bicarbonate (HCO_3_)	6.0 mmol/L	18.9 mmol/L	23-26 mmol/L
PCO_2_	15.2 mmHg	26.2 mmHg	35.0-40.0 mmHg
Urine acetone	Positive	Positive	Negative

A diagnosis of DKA, secondary to UTI, was made. Initially, the patient was managed with two 500 mL of normal saline, each infused with 20 mEq of potassium chloride (KCl) at 100 mL/hour. The fluid was titrated with urine output, which was 350 mL/ day initially. Regular insulin of 0.1 U/kg/hour was given by infusion pump. 100 MEq of intravenous (IV) sodium bicarbonate (NaHCO_3_) and antibiotic coverage were given for UTI. Her insulin infusion was titrated with a level of K+ and random blood sugar. Her insulin infusion was titrated according to potassium and blood glucose levels. We withheld insulin infusion for four hours when the K+ level dropped below 3 mmol/L, and 50 mL of 50% dextrose was given when the blood sugar level was below 150 mmol/L. Her urine output decreased to 100 mL/six hours with a rise in potassium levels following the next day of treatment, which had to be managed by HD. Following HD, she had one episode of hypoglycemia with a random blood sugar of 2.3 mmol/L, for which she was given 50 mL of 50% dextrose. Following the initiation of the meal, she was maintained on a continuous insulin infusion and was administered subcutaneous insulin before each meal. Her insulin infusion was kept at 0.05 U/kg/hour afterward. She was planned for alternate-day dialysis to prevent fluid overload and uremic complications. She was transferred from the intensive care unit (ICU) to the nephrology ward once the acidosis had been treated, and she was then successfully discharged.

## Discussion

The management principles of DKA in a patient with end-stage renal disease (ESRD) on MHD is complex and determined by various factors. An intriguing problem occurs when individuals with ESRD receive HD. DKA is characterized by hyperglycemia, hyperkalemia, and acidosis, efficiently treated by HD. The diagnosis of DKA during the initial post-dialysis period, however, may be compromised by this resolution. The improvement in laboratory results could misrepresent stability and lead medical professionals to ignore the potential of DKA. Clinicians must constantly look for potential problems and carefully monitor patients for new or deteriorating symptoms following HD. The best results for these individuals depend on prompt identification and adequate care. Each patient should be tailored according to volume status, serum glucose level, serum osmolality, serum potassium level, and general condition. Unlike the general population, it is difficult to generalize the treatment of DKA in anuric patients. The following recommendations are drawn from the literature we reviewed. 

Hyperosmolar state

Pontine myelinolysis and cerebral edema are the most critical fatal complications that can be prevented by adequately managing the hyperosmolar state. Hyperglycemia-induced osmotic diuresis is absent in an anuric patient, so they rarely present with dehydration and shock [8]. Most patients, although not all, present with low serum sodium levels in conjunction with hypertonicity. In this case, the patient showed improvement with insulin infusion, accompanied by careful blood glucose monitoring, electrolyte levels, and overall volume status [8]. Sometimes, emergency HD may be needed if the initial presentation is associated with extreme hyperglycemia, hypertonicity, and a low serum sodium level. This, however, does not mean that we can perform early dialysis without proper indications, which if done can lead to cerebral edema due to a rapid decrease in serum osmolality [8,11]. The recommended reduction rate of serum glucose level varies, but most believe it to be around 50-75 mg/dl/hour [8]. In our case, fluid therapy was initially titrated according to the urine output and was balanced with the risk of developing pulmonary edema.

Fluid management

Fluid management is a crucial step in managing DKA. A patient with normal renal function is expected to have around 5-8 L of fluid loss. Still, in an anuric patient on HD, the scenario is different because of the absence of loss of osmotic diuresis [8]. Thus, fluid replacement in DKA in CKD under MHD should include 250-500 mL boluses guided by the patient's volume status; in case of significant fluid overload, it should be managed by HD [11,12]. In a patient of DKA with CKD, unlike the general population, there will be no osmotic diuresis due to hyperglycemia, ultimately leading to the expansion of extracellular volume [13,14]. Cautious fluid replacement should be done, guided by the patient's volume status, to prevent pulmonary edema [8]. Lim et al. suggested using isotonic saline as a fluid of choice. Dextrose normal saline can be used if there is a drop in blood sugar levels [11]. 

Insulin therapy

The mainstay of treatment of DKA is insulin therapy, which decreases the serum glucose levels and synthesis of ketone bodies and corrects hyperkalemia [8,14]. An insulin infusion is preferred over the subcutaneous route as it is easier to titer [8]. As insulin is excreted renally, most studies recommend a dose reduction for patients with DKA and ESRD as there is a decreased rate of elimination of insulin due to loss of renal function [8,9]. The recommended infusion rate is 0.05 to 0.07 units/kg per hour to prevent rapid drops in blood glucose levels and osmolality changes [8]. The rate of infusion can also be guided by a reduction of serum ketone level, which should be around 0.5 mmol/h; a decrease of serum glucose level, which should be about 50-75 mg/dl per hour; and an increase in serum bicarbonate level, which should be around 3 mmol/hour [8]. Vigilant monitoring of capillary blood glucose is recommended as patients with DKA in ESRD have a higher incidence of hypoglycemic events and other adverse glycemic events than the general population [15]. Thus, a slower rate of insulin infusion was recommended by Kuverji et al. to prevent hypoglycemic episodes [12]. The patient initially received six days of insulin infusion at 0.1 U/kg/hour. This was followed by 0.05 U/kg/hour after dialysis until her glucose level was less than 200 mEq/L with correction of acidosis. She was started on subcutaneous regular insulin and titrated accordingly after she tolerated the oral intake of meals. She developed one episode of hypoglycemia following HD, which was corrected with dextrose. Hypoglycemia is common after HD, depending on the glucose content in the dialysate. 

Electrolyte disturbances

Hyperkalemia is a known complication in patients with hyperglycemia, even without acidosis. However, severe hyperkalemia is usually absent in a patient with intact renal function by increasing renal loss of potassium [14]. Diabetic patients under MHD have severely impaired renal function, which increases the risk of fatal hyperkalemia associated with hyperglycemia [14]. Seddik et al. recommended not replacing potassium in a patient with DKA with ESRD on HD, even with the evidence of biochemical hypokalemia, as the total body potassium levels remain high due to the inability to excrete potassium renally [8]. Patients with a serum potassium level of >5.5 mmol/L should be on continuous cardiac monitoring; also, they may require emergency HD and insulin therapy for its management. Correction with 40 mmol/L of KCL should be done to reach the target potassium level of >3.5mmol/L in patients with a serum potassium level of <3.5 mmol/L [8,11]. Potassium replacement, if done at initial presentation without laboratory evidence of hypokalemia, might be fatal in the case of DKA with abnormal renal function. Insulin therapy alone can be used to treat the majority of hyperkalemia associated with DKA, as studies have concluded that insulin deficiency is its primary cause [14]. As the potassium level was 3.5 mmol/L in our case, 40 mEq/L of KCl was given with normal saline infusion at 100 ml/hour to prevent hypokalemia. The potassium level was measured every four hours to monitor hypokalemia due to insulin and possible hyperkalemia that may require immediate management. HD can be started when the potassium level crosses 5.5 mmol/L despite insulin therapy. 

Metabolic acidosis 

Severe metabolic acidosis can be seen at initial presentation in an anuric patient. Seddik et al. recommended emergency HD for correcting acidosis but suggested against bicarbonate administration [8]. Lem et al. stated that ESRD patients are accustomed to acidosis and, therefore, did not recommend bicarbonate therapy [11]. In chronic renal failure patients, there is a lack of excretion of metabolizable acids and a lack of regeneration of bicarbonate to counteract the acidosis. Therefore, bicarbonate supplementation must be started when the pH is less than 7.2 or HCO_3_ is 20-22 mmol/L as the acidosis increases protein catabolism.

Precipitating factors

Identifying precipitating factors, such as missed insulin dosing and infection, is essential in managing and preventing the recurrence of DKA [11]. In our case, plenty of pus cells in urine analysis and high lactate levels in arterial blood gas (ABG) analysis warranted using parental piperacillin and tazobactam for probable sepsis. Urine culture showed a growth of *E. coli*, suggesting UTI, which is the precipitating factor of DKA.

## Conclusions

Managing DKA in patients with CKD under MHD has challenges as it is an uncommon presentation. The management requires specific approaches due to the risk of volume overload and hyperkalemia in an individualized manner. Critical factors, such as hyperosmolar state, insulin therapy, electrolyte disturbances, and metabolic acidosis, should be attended to carefully. Identifying and promptly managing inciting events, such as infection and dehydration, is crucial for the proper management of the condition and prevention in the future.

Due to the lack of literature on this topic, this case report highlights the importance of additional research and formulation of specific management protocols for this condition, which can lead to improvement in management strategies and reduced complication rates in this unique but challenging medical scenario.
